# Middle Meningeal Artery Embolization in Chronic Subdural Hematoma: Implications of Pathophysiology in Trial Design

**DOI:** 10.3389/fneur.2020.00923

**Published:** 2020-08-27

**Authors:** Pouria Moshayedi, David S. Liebeskind

**Affiliations:** Department of Neurology, UCLA Comprehensive Stroke Center, University of California, Los Angeles, Los Angeles, CA, United States

**Keywords:** endovascular treatment, embolization, interventional neuroradiology, chronic subdural hematoma, middle meningeal artery

## Abstract

**Background:** Chronic subdural hematoma (cSDH) is a debilitating condition with a high rate of recurrence after surgical evacuation.

**Summary:** This review is focused on middle meningeal artery (MMA) embolization to treat cSDH. We discuss the underlying pathophysiology of chronic subdural hematoma and how cessation of arterial flow may resolve a venous hemorrhage. We also present the current evidence for MMA embolization and the roadmap for future trials.

**Conclusion:** Frequent multimodal imaging and cSDH sampling would enable us to understand mechanisms of MMA embolization in cSDH treatment and therefore improve our ability to offer MMA embolization to the eligible population.

## Chronic Subdural Hematoma: Clinical Presentation and Treatment Options

Chronic subdural hematoma (cSDH) is a collection of blood, blood degradation products, and fluids encapsulated in the potential space between the arachnoid and the dura known as the subdural space. cSDH is relatively common and it has increased in frequency in parallel to an increase in the aging population. It is estimated to occur in 17–20 patients per 100,000 population per year (1, 2), which is twice the frequency of aneurysmal subarachnoid hemorrhage (3). It commonly presents with unspecific symptoms of cognitive or behavioral changes. Its insidious progression poses a diagnostic challenge that leads to its “chronic” discovery. A 6–12-months mortality of 30% (4) testifies to the high disease burden.

Subdural hematoma occurs spontaneously or as result of trauma. Use of antiplatelets or coagulopathy (pharmacologic or due to hepatic failure) increases the propensity for hemorrhage (5). Surgical treatment in cases of cSDH with significant mass effect (usually >10 mm blood thickness or >5 mm midline shift) is indicated and it is commonly performed through a single burr-hole or craniotomy drainage and irrigation (6). However, between 9.4 (7) and 30% (8) of cases will experience re-accumulation of hematoma. Among patients with a one-time SDH recurrence, a subsequent hematoma recurrence has been observed in nearly half (9). Factors increasing risk of recurrence include diabetes, liver dysfunction, use of anticoagulants, and post-operative residual air in the subdural space (10, 11).

## Chronic Subdural Hematoma: Pathophysiology

There have been observational speculations about the pathophysiology of cSDH recurrence. The dominant theory revolves around rupture of bridging veins traversing from the brain to draining dural sinuses within the subdural space (12), but there are several characteristics of cSDH that argue for a more complex process: (1) cSDH takes several weeks to grow (13) that is longer than expected from a venous source of bleeding; (2) cSDH often extends across the cerebral convexities away from medial draining sinuses where bridging veins are predominantly located; and (3) acute hemorrhage is only observed in 9% of patients with growing cSDH (14), suggesting acute hemorrhage is not the etiology for a majority of cases. Alternative explanations have centered on a self-propagating cycle of inflammation, angiogenesis, exudation, and hemorrhage, which is described below.

cSDH occurs in a potential space between the brain and the dura populated with “dural border cells” (15). Initial hemorrhage occurs within the subdural space following a minor trauma in the context of increased traction from a shrinking aging brain. Hemorrhage leads to proliferation of dural border cells (16). In 21% of cases with acute SDH a sustained state of inflammation ensues leading to evolution of cSDH (17): influx of inflammatory cells to the injured dural border cells layer promotes proliferation of the cells into forming new membranes. Disruption of dural border cell layer leads to deposition of collagenous material to form the fibro-cellular connective tissue (18) in a process mirroring wound repair. Disrupted dural border cell layer subsequently reorganizes into the outer and the inner membranes, which are adjacent to dura and arachnoid layers, respectively (19). The inner membrane is a fibro-collagenous tissue with minimal vasculature or inflammation that does not contribute to cSDH growth (19), but in contrast, the outer membrane has been populated with neutrophils, lymphocytes, macrophages, eosinophils, and newly-formed vessels (20). Some studies have associated the angiographic “wispiness” of distal MMA branches with neovascularization in this layer (21). The new blood vessels have thin-walls with thin or no basement membrane and are devoid of smooth muscle cells or pericytes (20, 22) allowing continuous exudation of plasma and RBC into the subdural space (19, 22). Fragility of blood vessels in the outer membrane has been associated with intermittent acute bleeding in cSDH manifested as CT hyperdense foci (20).

**Figure 1:**
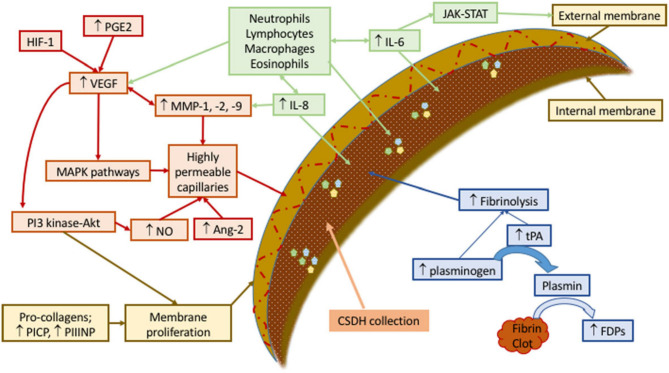
Schematic representation of mechanisms involved in development and sustenance of cSDH. Contributing factors have been labeled as green (recruiting inflammatory cell), red (forming permeable capillaries), brown (forming inner and outer membranes), and blue (ongoing hemorrhage due to fibrinolysis). Ang, angiopoietin; FDPs, fibrin/fibrinogen degradation products; HIF, hypoxia-inducible factor; IL, interleukin; JAK-STAT, Janus kinase-signal transducer and activator of transcription; MAPK, mitogen-activated protein kinase; MMP, matrix metalloproteinase; NO, nitric oxide; PGE, prostaglandin E; PI3-Akt, phosphatidylinositol 3-kinase-serine/threonine kinase; PICP, procollagen type 1; PIIINP, procollagen type 3; tPA, tissue plasminogen activator; VEGF, vascular endothelial growth factor. Reproduced from (24) under the terms of *the Creative Commons Attribution 4.0 International License* (http://creativecommons.org/licenses/by/4.0/).

Secretion of pro-inflammatory factors, such as vascular endothelial-derived growth factor, tissue plasminogen activator, angiopoietin-2, matrix metalloproteinases, tumor necrosis factor-α, interleukin (IL)-6, IL-8, thrombomodulin, and basic fibroblast growth factor (23), by the outer membrane into the subdural space fuels ongoing inflammation in a contained collection of blood, blood degradation products, and exudated fluids. The relevant question is how occlusion of middle meningeal artery (MMA) leads to the resolution of a self-perpetuating contained sac of inflammation in the subdural space. Figure 1 summarizes the factors contributing to the formation of cSDH.

## Middle Meningeal Artery Embolization as a Treatment Option for cSDH

cSDH recurrence is not uncommon. Surgical drainage of cSDH fails to cure in 9.4–30% (7) of patients. Some undergo repeat surgical treatment but if surgical treatment fails once, further recurrences are more common and some estimate the rate to be as high as 46% (9). Remaining options are limited and include peritoneal shunt, application of Ommaya reservoir, or endoscopic drainage and debridement (6, 10), none of which are proven to be effective.

Komiyama first introduced MMA embolization as a treatment option for recurrent cSDH in 1994 (25). Several case series have then been published on utilization of MMA embolization to treat cSDH refractory to surgical drainage. In a review, 21 cases of cSDH failed 1–7 times surgical drainage were successfully treated with MMA embolization (9). In a larger report of 72 consecutive patients (26) MMA embolization was performed in cSDH as a sole therapy or an adjunct treatment prior to surgical drainage in 27 and 45 patients, respectively. The success rate for MMA treatment was remarkable: There was no failure for MMA embolization as the sole treatment, and a 2.2% failure rate was reported when MMA occlusion was combined with surgical treatment. A comparison with historical control patients treated only with surgery revealed MMA embolization outperformed surgical drainage (*p* < 0.001).

MMA embolization has also been the subject of case-controlled studies. In a meta-analysis of 8 case-control studies (27), the rate of treatment failure in MMA embolization and conventional surgical treatment was 2.1 vs. 27.7%, respectively (OR 0.87, 95% CI 0.026–0.292, *p* < 0.001). In other more recent systematic reviews, the failure for cSDH patients undergoing MMA embolization was 4.1 and 2.4% for primary and recurrent cases, respectively (28). The need for surgical rescue in patient undergoing MMA embolization was 2.7% (29). MMA embolization had a 26 and 20% risk reduction for cSDH recurrence and surgical intervention, respectively (29). The rate of complications for MMA embolization was 1.2% (29). Given those promising results, a randomized clinical trial (ChiCTR1800018714), three parallel assignment open label studies (NCT04065113, NCT04095819, NCT04270955) and a single arm open label study (NCT03307395) are recruiting patients to further study MMA embolization in cSDH. In addition, they are more clinical studies planned but not yet commenced (NCT04272996, EMBOLISE NCT04402632, and EMPROTECT NCT04372147).

## How Does Blocking Arterial Blood Flow Cure Subdural Hematoma?

MMA embolization has proven effective in treating cSDH in non-randomized case-control studies. However, it is important to understand the mechanism for cessation of arterial blood supply to treat a hemorrhage that is venous in nature. Understanding mechanisms of therapeutic effects will enable us to offer MMA embolization to the eligible population and improve design of future randomized trials to provide high quality evidence for effectiveness of MMA embolization to treat cSDH.

**Figure 2:**
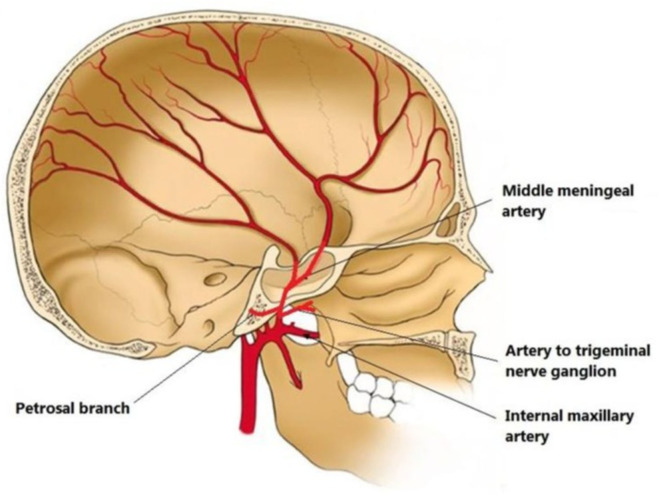
Illustration of middle meningeal artery (MMA) anatomy originated from internal maxillary artery and coursing in the inner skull. From (30) distributed under *Creative Commons Public Domain Mark 1.0*.

MMA is a branch of the maxillary artery, which itself is derived from the external carotid artery. It enters the skull through the foramen spinosum, courses through dura and divides into frontal and parietal branches (Figure 2). The MMA, together with anterior meningeal artery and posterior meningeal artery, supplies the meninges. Therefore, the MMA supplies blood to cSDH located in the mid-anterior to mid-posterior cerebral convexity.

It is important to mention anastomoses of distal MMA branches with ophthalmic artery via the recurrent meningeal artery, and with posterior auricular artery supplying facial nerve, since inadvertent leaking of embolizing particle materials can cause ophthalmic nerve and facial nerve injuries, respectively (31). An ophthalmic artery origin of MMA has been observed in 13.8% of patients with cSDH compared with 0.7% of patients with epistaxis selected as control (32). Whether it is causative or adaptive in recurrent cSDH, or simply a co-occurrence, an ophthalmic origin of MMA prohibits embolization as a treatment for cSDH. Another technical consideration is the choice of embolizing material: majority of studies have utilized polyvinyl alcohol (PVA) particles (14) and few have used liquid embolic material. There are theoretical advantages for particles or liquid materials: particles have more distal penetrance and block most distal branches that may receive collateral perfusion from other arteries than the MMA, however, particles are not opaque and they are hard to visually track. On the other hand, small size of microcatheters only allows limited volume delivery of particles, while larger amounts of liquid embolic agents can be injected under constant pressure. Clinical studies are warranted to compare efficacy of different embolizing materials in treating cSDH.

Utilization of the MMA in treating cSDH has also been suggested by the observation that MMA appears engorged in cSDH (33). As reviewed above, collection of cSDH is caused by an active biologic process of exudation and formation of loose vasculature prone to spontaneous bleeding. Therefore, occlusion of the MMA does not simply block blood pumping into the subdural space, but rather affects the complex biology of inner and outer membranes lining the cSDH cavity. cSDH CT images obtained after MMA embolization have shown contrast material enhancement of dura, capsular membrane, septations, and subdural hematoma fluid, suggesting a continuous vasculature between the cSDH membranes and the MMA (34). It is therefore expected that MMA embolization causes ischemia in the outer membrane, as well as the inner membrane, which leads to resolution of cSDH.

In order to understand the pathophysiology of cSDH resolution following MMA embolization it is helpful to discuss a similar pathology treated with arterial embolization: hypervascular intracranial tumors and their pre-surgical embolization. Arterial embolization is used as an adjunct to surgical resection to diminish intraoperative hemorrhage and decrease tumor size. It is typically used in hypervascular tumors, such as meningioma, located in deep cranial locations like skull base (35). Histopathologic assessment of embolized tumors have revealed that arterial embolization induces cellular dissociation and ischemic cellular changes such as cell shrinkage, nuclear pyknosis, and karyorrhexis (36). There are areas of confluent necrosis and micronecrosis (37), as well as apoptosis in the perinecrotic areas (37). Polymorphonuclear infiltration follows and leads to the formation of perivascular cuffing and inflammatory reaction in the surrounding area (38). Some degrees of cell proliferation and neo-angiogenesis follow inflammation (37). Super selective embolization of tumor feeding artery has been associated with 3.2% (38) to 5.1% (39) rates of tumor hemorrhage.

In contrast to pre-surgical tumor embolization, tissue specimens are not readily available in cSDH after MMA embolization. It has been suggested, however, that occlusion of MMA leads to ischemia in inner and outer membranes that subsequently impairs their biological role in sustaining cSDH. Beyond this speculation we do not know the details of biochemical cascades in cSDH and surrounding membranes following MMA embolization. We expect that a higher metabolic state of surrounding membranes makes them susceptible to ischemia and allows MMA embolization to selectively eliminate inner and outer membranes following necrosis and apoptosis. Cell death inevitably causes inflammation, cell proliferation, and neovascularization, but these processes just as in tumor embolization (38) do not usually cause swelling and hemorrhage in the cSDH collection sac (26, 27, 40, 41). In cSDH membranes surviving MMA embolization, ischemia probably impairs active processes of cell proliferation, angiogenesis, and secretion. These would halt secretion into the cSDH and allows the resorption to supersede and resolve cSDH. All of the above explanations are speculative but there are aspects of trial design that can help in validating probable processes of cSDH resolution.

## How Can Clinical Trial Design Inform MMA Embolization?

Repeated brain MRI allows for tracking changes in the thickness and composition of surrounding membrane, as well as the inner sac composition and size following MMA embolization. Relative changes in inner vs. the more vascular outer membrane will inform on their susceptibility to ischemia. Contrast-enhanced MRI allows for evaluation of blood-brain barrier integrity that could be compromised by inflammation or ischemia. Monitoring the possible enhancement of surrounding membranes informs us of the baseline permeability within these membranes and the changes following MMA embolization. This approach has been implemented and a higher enhancement of cSDH membranes have been correlated with a shorter interval for hematoma recurrence (42). Positron emission tomography (PET) has been previously utilized in cSDH and fluorodeoxyglucose uptake attributed to high metabolic activity (43). The use of PET in trials may help to track metabolic activity following cessation of MMA blood flow. Those patients with a subdural shunt or Ommaya reservoir can also provide us with a valuable opportunity to sample the cSDH content before and after embolization to analyze levels of inflammatory factors (e.g., vascular endothelial-derived growth factor, tissue plasminogen activator, angiopoietin-2, matrix metalloproteinases, tumor necrosis factor-α, IL-6, IL-8, thrombomodulin, and basic fibroblast growth factor) and blood degradation products and thereby record the sequence of events following MMA embolization. The current studies on MMA embolization and cSDH have not reported such information (26, 27, 40, 41) and future studies may help to shed light on the pathophysiology of MMA embolization in cSDH resolution.

Patients with cSDH are either (1) surgically naive, (2) surgically failed, or (3) receiving MMA as adjunctive post-surgical modality. It is also important to choose a homogenous population for future trials since each of those sub-groups of patients have different propensities to fail treatment and re-accumulate hematoma. Progression or reduction of hematoma measured at certain time point has been commonly used as the primary outcome, but in a patient-centered approach it is important to include parameters such as time to restart antiplatelets or anticoagulants indicated due to other cerebrovascular or cardiovascular conditions. A patient's clinical response to treatment should be independently assessed as a trial endpoint. Since cSDH does not often cause focal neurologic symptoms, neurocognitive assessments may be used to track patients' clinical improvement. Imaging endpoints may include cSDH size, change in cSDH, or change in the degree of membrane enhancement following MMA embolization.

Given heterogeneity of the studied population, as well as differences in imaging modalities and measurement techniques, development of standardized methods of patient selection, and imaging analyses is recommended, to facilitate sample size estimation and statistical meta-analysis. There is a need for unbiased non-industry funded trials to impartially assess effectiveness and elucidate underlying mechanisms for MMA embolization in treating cSDH.

## Conclusion

MMA embolization has been very effective in treating cSDH, but limited understanding of cSDH cure mechanisms curtails our ability to offer MMA embolization to the eligible population and improve the design of future randomized trials. By introducing frequent multimodal imaging and use of contrasted studies, as well as cSDH sampling, we may be able to monitor changes following MMA embolization and provide high quality evidence for the effectiveness of MMA embolization.

## Author Contributions

PM and DL: conception, design, analysis, interpretation of data, critically revising the article, reviewed submitted version of manuscript, and administrative/technical/material support. PM: acquisition of data and drafting the article. DL: approved the final version of the manuscript on behalf of all authors and study supervision. All authors contributed to the article and approved the submitted version.

## Conflict of Interest

DL is consultant to Cerenovus, Genentech, Stryker, and Medtronic as Imaging Core Lab. The remaining author declares that the research was conducted in the absence of any commercial or financial relationships that could be construed as a potential conflict of interest.

## Abbreviations

cSDH: Chronic subdural hematoma
IL: interleukin
MMA: middle meningeal artery.
